# Is unilateral lower leg orthosis with a circular foot unit in the treatment of idiopathic clubfeet a reasonable bracing alternative in the Ponseti method? Five-year results of a supraregional paediatric-orthopaedic centre

**DOI:** 10.1186/s12891-018-2160-1

**Published:** 2018-07-18

**Authors:** N. Berger, D. Lewens, M. Salzmann, A. Hapfelmeier, L. Döderlein, P. M. Prodinger

**Affiliations:** 1 Klinikum rechts der Isar der Technischen Universität München, Munich, Germany; 2 Behandlungszentrum Aschau im Chiemgau, Aschau, Germany; 3 Institute of Medical Informatics, Statistics and Epidemiology, Technical University Munich, Munich, Germany

**Keywords:** Clubfoot, Ponseti, Brace, Unilateral Orthosis, AFO, Pohlig Baise articulated lower leg Orthosis

## Abstract

**Background:**

In the Ponseti treatment of idiopathic clubfoot, children are generally provided with a standard foot abduction orthosis (FAO). A significant proportion of these patients experience irresolvable problems with the FAO leading to therapeutic non-compliance and eventual relapse. Accordingly, these patients were equipped with a unilateral lower leg orthosis (LLO) developed in our institution. The goal of this retrospective study was to determine compliance with and the efficacy of the LLO as an alternative treatment measure. The minimum follow-up was 5 years.

**Results:**

A total of 45 patients (75 ft) were retrospectively registered and included in the study. Compliance with the bracing protocol was 91% with the LLO and 46% with the FAO. The most common problems with the FAO were sleep disturbance (50%) and cutaneous problems (45%). Nine percent of patients experienced sleep disturbance, and no cutaneous problems occurred with the LLO. Thirteen percent of patients being treated with an FAO until the age of four (23 patients; 40 ft) underwent surgery because of relapse, defined by rigid recurrence of any of the components of a clubfoot. Fourteen percent of patients being treated with an LLO (22 patients; 35 ft), mostly following initial treatment with an FAO, experienced recurrence.

**Conclusion:**

Changing from FAO to LLO at any point during treatment did not result in an increased rate of surgery and caused few problems.

**Electronic supplementary material:**

The online version of this article (10.1186/s12891-018-2160-1) contains supplementary material, which is available to authorized users.

## Background

The Ponseti method [1] is universally accepted as the gold standard for correcting idiopathic clubfoot. It involves serial manipulation and casting of the feet, mostly combined with an Achilles tenotomy, followed by the use of a foot abduction orthosis (FAO) to maintain the correction. This orthosis, which holds the foot in external rotation and dorsiflexion, must be worn for 23 h a day for 3 months, and for at least 10 h per night for an additional 3–4 years [2, 3]. The most common models follow the design of Denis Browne [4, 5], employing a rigid middle bar, high-top shoes, and maintaining the affected foot in up to 70° external rotation (non-affected foot: 30–40°) and 10° dorsiflexion. If the protocol is correctly maintained, recurrences needing surgery are reported to be around 12% [6–8].

However, parental non-compliance with the use of FAO during the course of treatment is an often reported problem [9–13]. Although it is well known that non-adherence to the bracing protocol results in elevated odds of recurrence (5- to 17-fold higher [6, 11, 14]), non-compliance is as high as 34–61% [3, 9, 11, 14–17].

To date, existing literature has identified low income and low educational levels as predicting factors for non-compliance [12, 16, 18]. Reasons given by parents for not using the brace are prolonged crying, disturbed sleep [10, 17, 19], and cutaneous problems such as skin irritation, blisters, and pressure sores [9, 18, 20].

In recent years, efforts have been made to develop more comfortable braces. Dynamic braces allowing a certain range of motion have shown promising results [9, 20, 21]. However, existing designs of ankle–foot orthoses have not yet provided an alternative to bracing because of the reported high recurrence rates (31% [22] and 83% [23]). George et al. [22] built an above knee orthosis, which consisted of three parts, shoe (sandal with laces), angled metal bar, and leg straps. The orthosis could be picked from the shelf and assembled as required. Janicki et al. [23] used a standard AFO consisting of a one piece plastic half-tube that was applied to the dorsal side of the leg and ended below the knee. Foot and shank were fixed with Velcro straps. The authors stated that their orthosis could not control abduction of the foot, which is required to stretch the medial soft tissues.

After completing Ponseti casting, our patients are generally provided with a standard FAO. If problems occur and cannot be solved, we provide the patient with a custom-made unilateral lower leg orthosis (LLO). In this retrospective study, we aimed to (1) evaluate compliance rates with FAO and LLO treatment, and (2) gain a first-hand impression of the efficacy of LLO in avoiding clubfoot recurrence.

## Methods

Inclusion criteria for the present study were the diagnosis of idiopathic clubfoot, complete documentation, a minimum bracing period of 3 years after completion of casting (or less, if an operation was performed within the bracing period), and a minimum age at follow-up of 5 years.

**Fig. 1:**
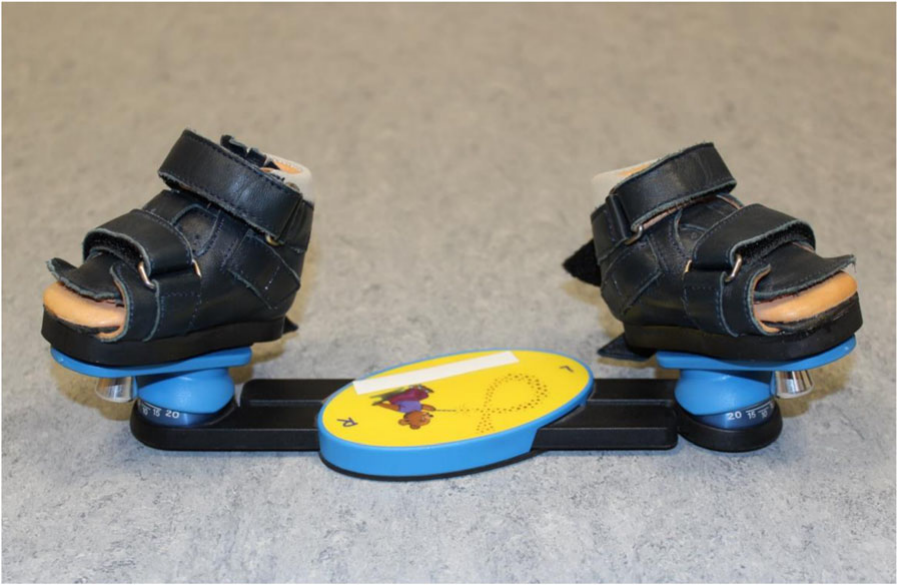
The foot abduction brace (FAO)

After completing the Ponseti series of castings, children were routinely provided with a standard foot abduction orthosis according to Denis Browne. High-top leather sandals closed by Velcro straps could be attached separately to a rigid middle bar (Fig. 1).

At each visit, we asked the parents about difficulties in complying with the bracing protocol. If the brace or orthosis was not put on for 23 h during the first 3 months of life and for at least 10 h per night until the end of the third year of life, it was regarded as non-compliance, and the reasons given by the parents were documented. The absence of signs of brace or orthosis use was also recorded.

Parents who reported problems with the FAO were specifically asked about the nature of the problem. Cutaneous problems were treated by adjusting the size and configuration of the standard shoes. If we detected a problem understanding the necessity for therapy, then the parents were advised thoroughly and encouraged to continue using the FAO. Parents who reported acceptance difficulties by the child, usually expressed by prolonged crying and problems sleeping through the night, were helped to implement a bed-time routine. If none of these measures were successful in re-establishing compliance with the bracing protocol, then we proposed an LLO. Some children were initially provided with the LLO if the treating doctor felt that the FAO might not be well tolerated.

### Construction of the LLO

**Fig. 2:**
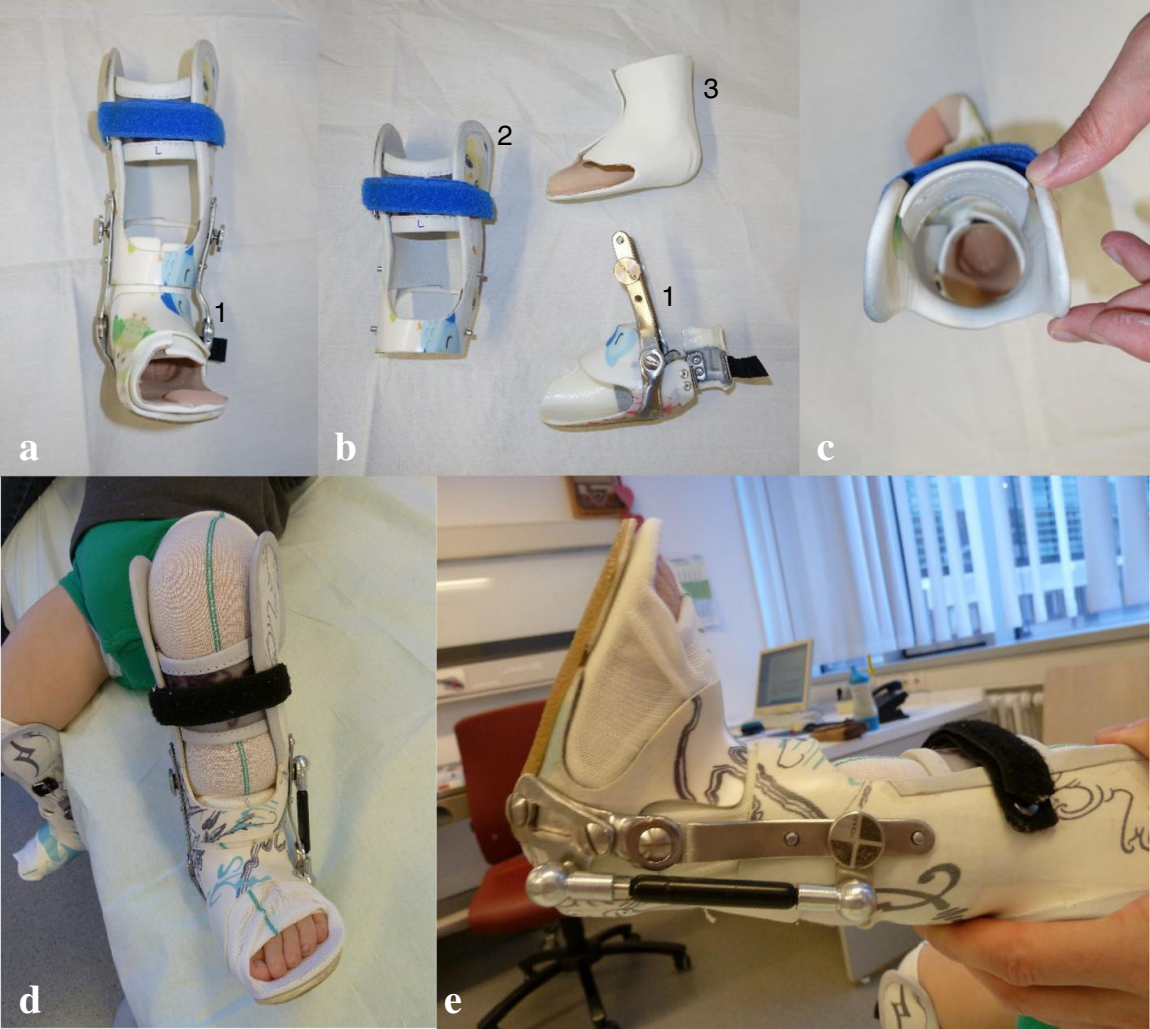
**a**-**e** The Pohlig lower leg orthosis (LLO). **a** + **b** Note the circular foot unit (1) closed by a heel cap as seen in **b**), the lower leg unit (2) and the inner liner made out of Tepefoam (3). The calcaneo-pedis block is held in 20° external rotation (see **a** and **c**) and 20° dorsal extension (see **e**). A mounted gas pressure spring to push into dorsal extension that can be adjusted, if desired, is also shown

The unilateral LLOs were custom-made with resin and carbon and were built in three parts following Baise and Pohlig’s 2005 design (Fig. 2a-c) [24]: a circular foot unit, a lower leg unit, and an inner liner made out of Tepefoam.

**Fig. 3:**
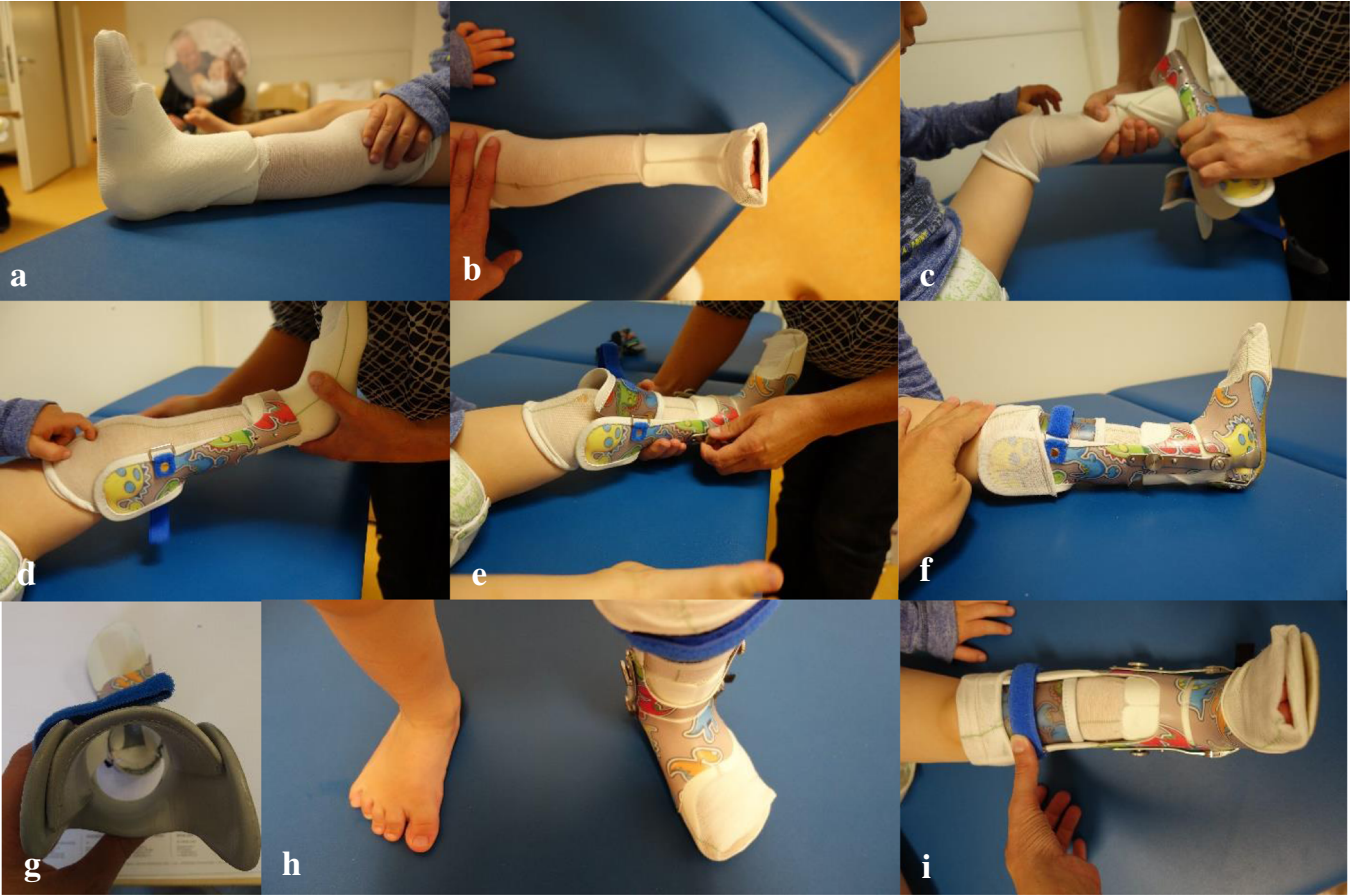
**a**-**i** Putting-on of the Pohlig lower leg orthosis: (**a** + **b**) A stocking is put on the leg before the Inliner is applied, then the stocking is pulled over the Inliner (**c** + **d**) Now the lower leg unit is slipped over the foot and fixed to the shank (**e**) The foot unit is slipped over the foot and fixed to the lower leg unit by screws. (**f**, **h**, **i**) The mounted orthosis fixes the foot in neutral dorsiflexion and 20° of external rotation. Further 5–10° of dorsiflexion are allowed by hinges when walking in the orthosis (**g**) Top view of the orthosis, demonstrating external rotation of the foot unit versus the lower leg unit

The foot unit follows the principles of the Calcaneus-Rotation-Ring type orthosis, described by Baise and Pohlig (2004) for the treatment of spastic clubfeet [25]. It fixes the subtalar joint in a valgus position. It does so by encasing the calcaneopedal unit [26], which is then everted in the subtalar joint line by a turning movement by the person who applies the orthosis. Once in place, the ring-like enclosure (completed by a heel cap) works like an external arthrodesis of the subtalar joint [25] (Fig. 2a-i ). The resulting hindfoot valgus is 10–15°. In the beginning, we externally rotated the foot against the knee joint line 40° and more, but this resulted in overcorrection. Hence, we reduced the rotation and now seek for an external rotation of 20° (Fig. 3a-d).

The foot unit is fixed to the lower leg unit by screws and hinges and allows a range of motion of 0–5-20° plantarflexion/dorsiflexion. Rotational stability of the orthosis in relation to the axis of the knee is mandatory to maintain the position and therefore the correctional capacity of the foot unit. This is achieved by mounting the lower leg unit with a combination of ear-shaped supports encompassing the femoral condyles at the proximal ending of the lower leg unit, working as a counter bearing against the rotational forces. The range of motion of the knee joint is not limited by those encompasses. Further stability is provided by a firm intake of the calf, realized by a Velcro-fixed resin cap above the tibial tuberosity that provides an intake working like a Sarmiento brace (Fig. 2a-e).

The inner liner or “Inliner” made out of Tepefoam works simultaneously as a pressure absorber and distributer of the correctional forces to the entire foot surface. Padding of Bisgaard’s region in the Inliner further prevents slipping of the heel. Besides, the Inliner alleviates the process of slipping into the foot unit (see also our online Additional file 1: Video S1).

These principles of construction allowed us to meet the demands of a post-Ponseti-brace: (1) stretching of the structures of the posterior and medial ankle and tarsal ligaments and musculo-tendinous units [2]; (2) allowing free kicking (and even walking), and thereby stretching of the gastrosoleus complex [27].

We did not use the term ‘ankle–foot orthosis’, because the orthosis also encompasses parts of the knee. Instead, we chose to introduce the term ‘lower leg orthosis’.

During follow-up, recurrence of the clubfoot position in one or more of its components (hindfoot varus, midfoot cavus, and forefoot adductus; defined on clinical basis) was documented and, if necessary, a second series of casting, re-tenotomy, or other invasive operative measures were performed.

### Statistics

The distribution of quantitative data is described by mean and range. Qualitative data is presented by absolute and relative frequencies. Corresponding hypothesis testing on group differences was performed by t-tests and Pearson’s chi-squared tests using exploratory two-sided 5% levels of significance. All statistical analyses were performed using R 3.4.2 (R Foundation for Statistical Computing, Vienna, Austria).

## Results

Between 2004 and 2011, we treated 177 children with clubfoot according to the Ponseti method. Forty-four patients were excluded from this study because of a non-idiopathic clubfoot. Ten patients had accompanying hip problems, so the Ponseti treatment had to be modified. Forty-nine patients were lost during follow-up and continued treatment with their local orthopaedic doctor. Of the remaining patients, 45 (75 ft) had a minimum follow-up of 5 years and were included in this study.

The mean age at follow-up was 8.2 years (range: 5.0–11.6 years). Fifty-three percent of the patients (*n* = 24) were pre-treated and referred to our clinic owing to persistence of the deformity. Children with pre-treated feet presented at our hospital at a mean age of 6.4 weeks. Fifteen children had unilateral clubfoot. The female to male ratio was 1:1.8. The mean initial Pirani score [28] at first presentation in our clinic was 4.9 (range: 1.0–6.0). A mean of seven casts (range: 1–12) were necessary. The foot with only one cast had an initial Pirani score of 1.5. Achilles tenotomy was performed in 88% of all feet. Complete initial correction was observed in all patients at the mean age of 14.6 weeks (range: 7.9–45.9 weeks). There was no residual cavus or adduction deformity and the minimum dorsiflexion capacity of the foot (measured in extended knee position) was 15° and the minimum passive abduction was 40° of the foot against the fixed talus as described by Ponseti [2].

### Compliance with the FAO and LLO

We registered problems with the FAO in 54% and with the LLO in 9% of treated patients (Table 1).

**Table 1:** Characteristics of patients in terms of compliance

Characteristic	Value
Non-compliance with FAO total, patients (% of patients; feet)	22 (54%; 34)
Non-compliance with FAO because of skin problems, patients (% of non-compliant patients; feet)	10 (45%; 14)
Non-compliance with FAO because of sleep disturbance, patients (% of non-compliant patients; feet)	11 (50%; 18)
Non-compliance with FAO for other reasons, patients (% of non-compliant patients; feet)	1 (5%; 2)
Non-compliance with LLO total, patients (% of patients; feet)	2 (9%; 4)
Non-compliance with LLO because of skin problems, patients (feet)	0
Non-compliance with LLO because of sleep disturbance, patients (feet)	2 (9%; 4)
Non-compliance with LLO for other reasons, patients (feet)	0

Forty-one children (70 ft) were initially treated with an FAO. Twenty-two children (54%) developed intermittent or lasting non-compliance with the bracing protocol. The most common problems were skin irritations/pressure sores (45%) and/or sleep problems (50%). Three out of ten patients suffering from serious cutaneous problems were successfully managed by changing to custom-made resin shoes, so the children could continue with FAO treatment.

Twenty-two children (35 ft) were treated with an LLO. Of those, 18 children (30 ft) were converted to LLO treatment as a consequence of discontent with the FAO at a mean age of 14.5 months (range: 2–34 months). Only two children (four feet; 11%) developed problems with the LLOs and showed prolonged crying and sleep disturbance (Table 1). No unresolvable pressure sores or complaints about the complexity of brace handling were noted with the LLOs.

### Efficacy of the FAO and LLO

**Fig. 4:**
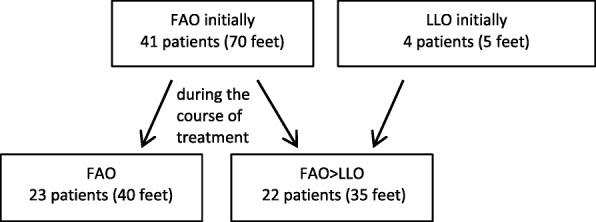
FAO: foot abduction brace. LLO: lower leg orthosis

Two groups of patients were defined with respect to the treatments: patients who remained on FAO treatment throughout (‘FAO only’; 23 patients, 40 ft) and patients who began treatment with LLO at any time-point during the treatment (LLO initially or following FAO treatment: ‘FAO > LLO group’; 22 patients, 35 ft) (Fig. 4 and Table 2). There was similar age at the beginning of cast treatment between the ‘FAO only’ group (mean 3.7 weeks, range 0.3–22.7) and the FAO > LLO (mean 5.1 weeks, range 0.3–40.3) patients (*p* = 0.527; *t*-test). There was minor difference between the groups in the number of pre-treated feet (FAO only: 38%, FAO > LLO: 44%; *p* = 0.6277), Pirani-score (mean: FAO only: 4,9; FAO > LLO: 4,8; *p* = 0.905), or mean follow-up period (FAO only: 8.0 years; FAO > LLO: 8.4 years; *p* = 0.597).

**Fig. 5:**
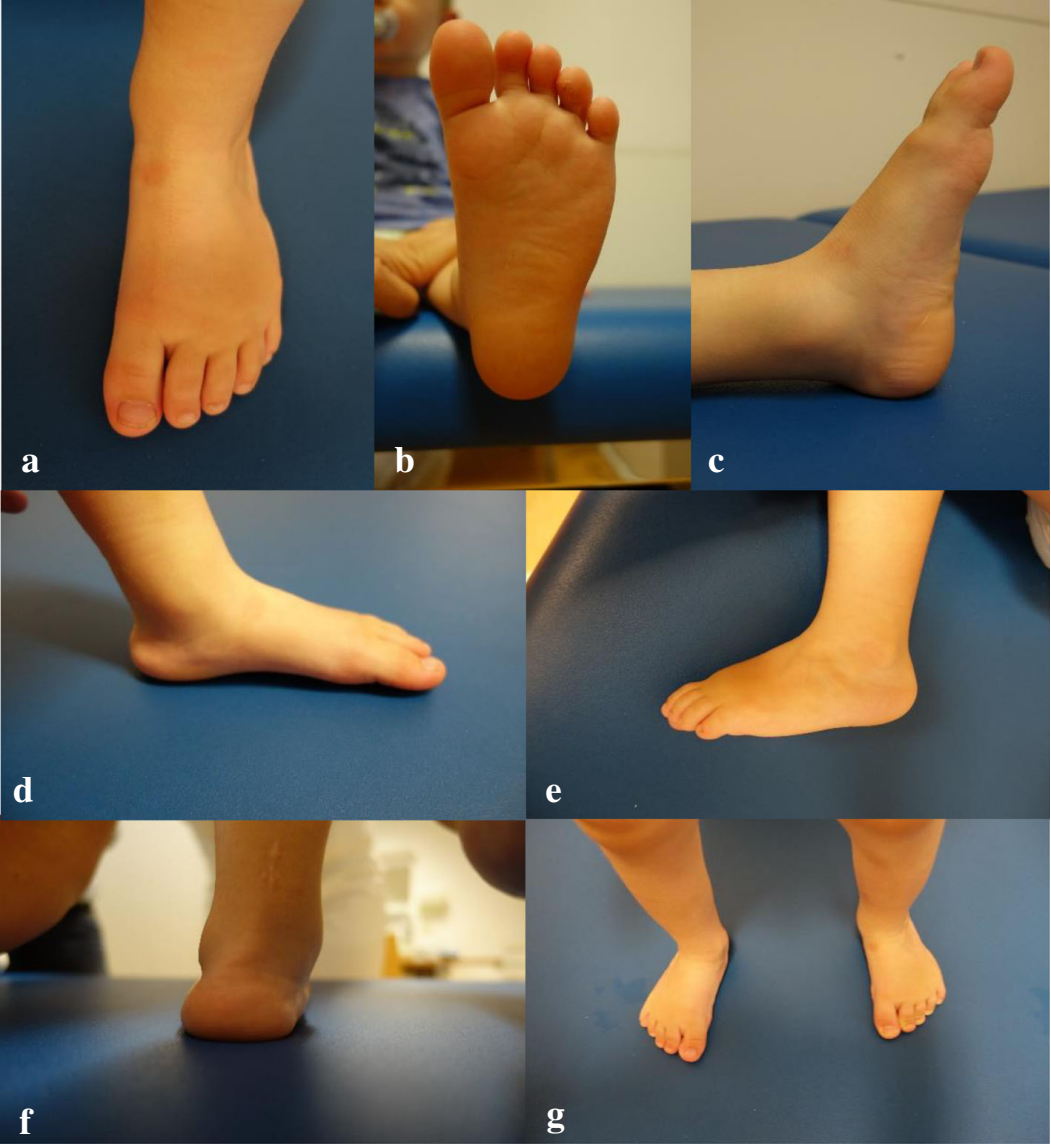
**a**-**g** This otherwise healthy boy (same boy as in Fig. 2) presented at birth with a congenital vertical talus at the right foot and a congenital clubfoot at the left side (Pirani score 6, stiff-soft). His treatment with LLO started right after removing the last casts. At the time the photographs were taken, he was 2.5 years old. (**a** + **b**) There is only minimal adduction of the greater toe and neutral orientation of the left forefoot (**c**, **d**). The heel is in slight valgus position (**f**). Residual from his deformity is a pronounced internal rotation of the left tibia (**g**)

**Table 2:** Characteristics of patients treated with a foot abduction brace (FAO) either exclusively until the end of treatment or follow-up (FAO only), or who were began on or switched to a lower leg orthosis (FAO > LLO)

Characteristic	FAO only	FAO > LLO
No. patients (feet)	23 (40)	22 (35)
Sex, female/male	10/13	6/16
Unilateral/ bilateral affected patients	6/17	9/13
Age at beginning of cast treatment, weeks (range)	3.7 (0.3–22.7)	5.1 (0.3–40.3)
Pre-treated feet, n	15 (38%)	16 (44%)
Initial Pirani score (range)	4.9 (1–6)	4.8 (1.5–6)
Mean wearing time, months per foot: FAO/LLO	40.9/−	8.1/30.7
Follow-up period, years (range)	8.4 (5.0–11.6)	8.0 (5.0–11.3)
Second series of casting/second tenotomy Achilles tendon, patients (feet)	1 (1)	0
Surgery because of relapse (retenotomy of Achilles tendon, soft tissue and bony procedures), patients (feet)	3 (3)	3 (3)

Four patients (five feet) started bracing therapy directly with an LLO. At the beginning of the bracing treatment, these patients had a mean age of 26.1 weeks (range: 13–46 weeks). They were all pre-treated with a minimum of four casts for a period of 0.5–9 months. At the time of presentation to our institution, their Pirani scores were 3, 4.5, 2 × 5, and 6 (Fig. 5).

Eighteen children (30 ft) changed to LLO treatment at a mean age of 14.5 months (range: 2–34 months). The mean wearing time of FAO per foot before switching to LLO was 8.1 months (range: 0–27 months) (see also Additional file 2).

The mean wearing time of LLO per foot (of all children treated with LLO, initially or following FAO treatment) was 30.7 months (range: 13.9–80.5 months) (Table 2). Three children (three feet; 14%) required additional surgery. Two children had a peritalar release performed at age 4.9 and 6.5 years. They had started with FAO treatment at the age of 2.6 and 2.3 months, respectively, and switched to LLO treatment owing to sleeping issues at age 3.9 and 6.9 months, respectively. Another child had received 9 months of casting elsewhere before being referred to us. This child first received an additional four casts at our institution; subsequently, bracing treatment was initiated directly with an LLO. Anterior tibial tendon transfer and calcaneal osteotomy followed at the age of 7 years.

In patients treated exclusively with the FAO (*n* = 23), the mean wearing time until end of treatment or relapse was 41.6 months (range: 28–49 months) per foot. One patient required a second series of casting. Three patients (three feet; 13%) developed recurrence requiring re-tenotomy of Achilles tendon and additional dorsal capsulotomy in one case (one patient, at age 13 months) or had major surgery (peritalar release; [two patients, at age 3.8 and 8.5 years]).

## Discussion

In this retrospective study, we investigated compliance with unilateral LLO or standard FAO. We also attempted to obtain some first impressions regarding the efficacy of the unilateral LLO. As this study took place at a specialized hospital, many patients were referred after pre-treatment at a relatively “old” age compared with patients normally seen at an outpatient clinic. Therefore, these patients may represent a negative selection regarding severity of the deformity or overall parental compliance.

Difficulties in maintaining the bracing-protocol for standard FAOs is well known in literature [29], with cutaneous problems reported in up to 45% of patients [9, 18, 20] and non-compliance rates of 34–61% [3, 9, 11, 14–17]. Non-compliance is usually defined as the interruption or discontinuation of the recommended scheme (23 h of bracing for the first 3 months, then 10–12 h per day until the age of 3–4 years). In a recent study by Goksan et al. [29], compliance with the orthosis was defined as fulltime brace use for 3 months and during sleep for ≥9 months. In this study, difficulties with the brace were encountered in 80% of affected children [29]. Comparatively, dynamic orthoses seem to result in fewer cutaneous problems (0–3.6%) and less non-compliance (7–38%), probably by reducing the lever on the heel and allowing more active movement [9, 20, 21].

The rate of non-adherence with the bracing protocol in our study was 9% with the lower leg orthosis, and 54% in children treated with a foot abduction orthosis. The most important problems reported with the FAO were problems sleeping through the night (50%) and skin problems (45%). With the LLO, only sleep disturbance was observed. In our study, non-compliance meant that patients did not wear the brace full-time for the advised time of three whole months and at least 10 h during night-time until the age of 3–4 years. Of the 18 patients who did not accept the FAO and probably would have discontinued the bracing protocol, 16 patients successfully continued with the LLO.

**Fig. 6:**
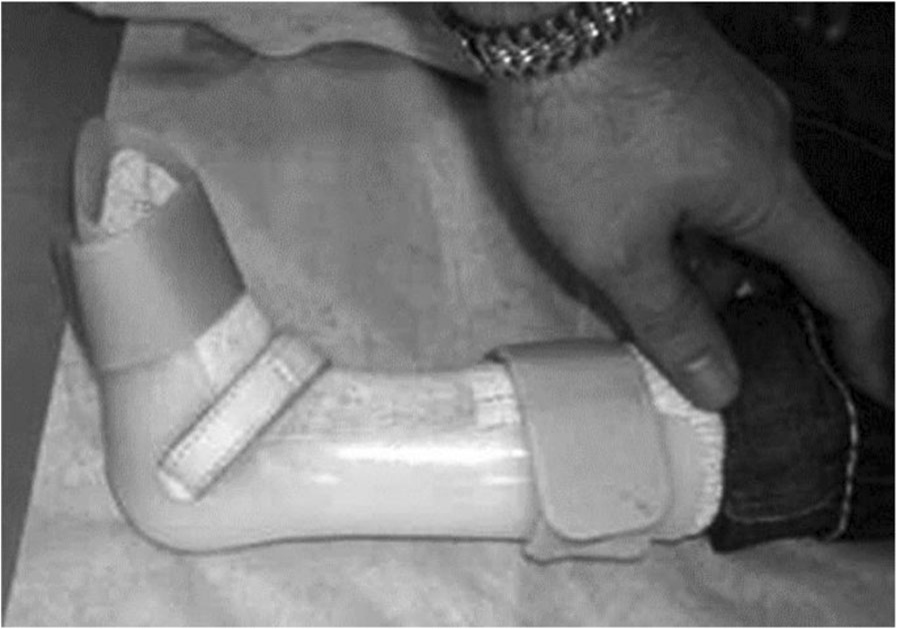
The orthosis used by Janicki et al. 2011, following the classic AFO design [23]

**Fig. 7:**
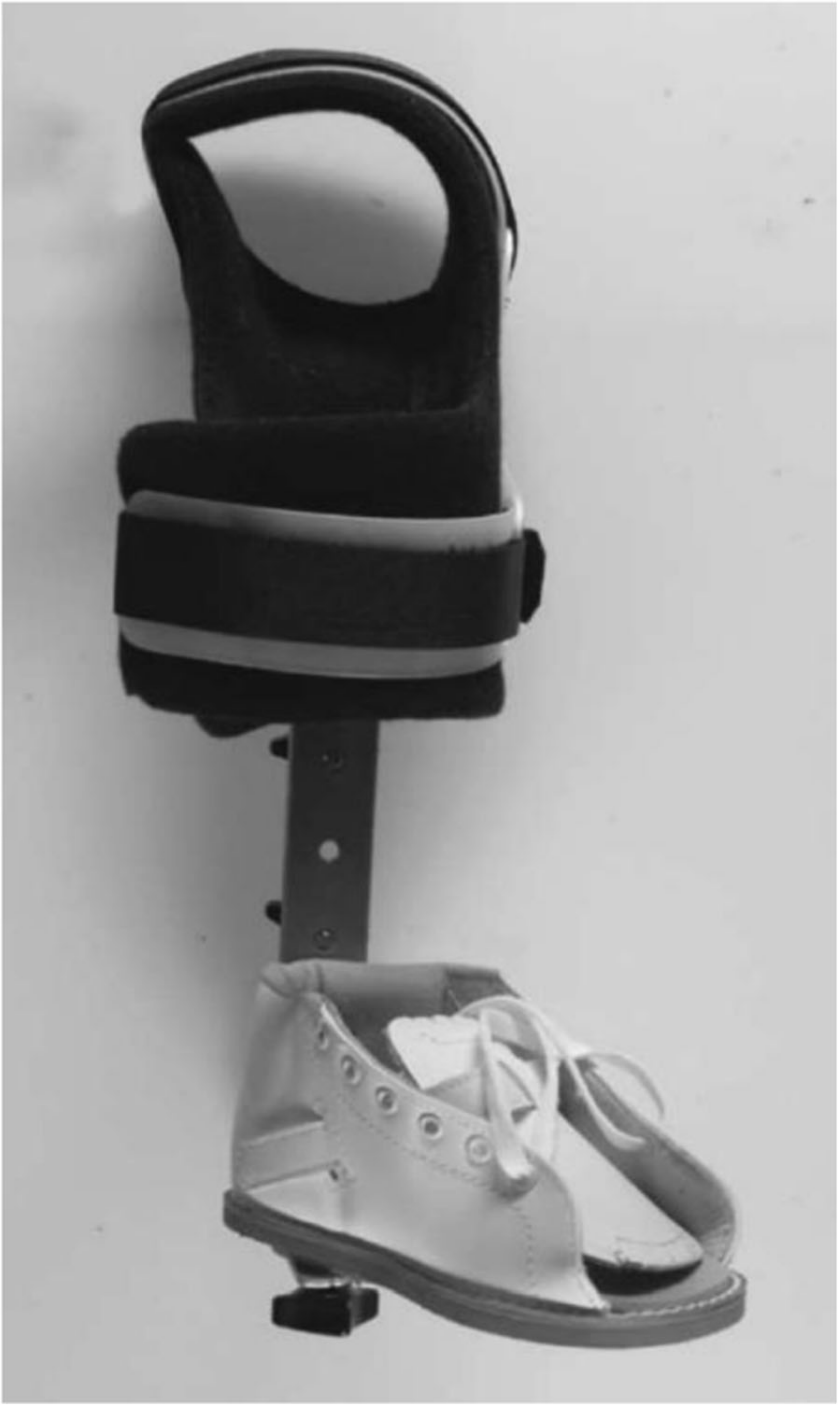
The orthosis used by George et al. 2011, using a modular system [22]

At present, it is of doubt whether an ‘ankle–foot orthosis’ can guarantee foot abduction or external rotation [3, 22, 23] representing major columns in treating clubfeet. To our knowledge, the literature has so far reported only two studies of post-Ponseti bracing with a unilateral ankle–foot orthosis. Janicki et al. investigated the use of a classic AFO fixed with Velcro straps [23] (Fig. 6), while George et al. presented a system consisting of a shoe fixed to a lateral bar spanning the knee at a 90° angle, fixed by straps at the shank [22] (Fig. 7). Although compliance was good (up to 85% [22]) with both versions, relapse was seen in 83% [23] and 31% [22] of feet, respectively, over follow-up periods of 60 (range: 50–72) [23] and 25 (range: 16–36) months [22]. Recurrence occurred after an average of 33.3 weeks (range: 4–76) [23]. To summarize, the results were not encouraging and both authors recommended not using the unilateral orthoses any further.

In the classic orthotic AFO design as used by Janicki et al. [23] the foot is fixed into a dorsally applied shell by Velcro straps (Fig. 6). To our experience, a foot can move in a plastic half-tube shell to a certain amount around its axis, and supination of the foot is quite easily performed. In our design of the articulated lower leg orthosis the foot is held in a full-contact custom-made circular encompassing, preventing any undesired movement of the foot once the heel cap is closed. The pressure in this circular holding is applied on a large scale and thereby prevents pressure sores.

Another problem in the classic AFO design is to maintain external rotation of the foot to the knee joint line. The rotational forces have to be antagonised by sufficient friction and abutment.

In the classic AFO design a counter bearing is missing. The articulated LLO works with a lateral support at the femoral level via condylar encompasses that transmit the rotational forces to the femoral condyles, whether in knee flexion or extension. This follows the same principles as the toe-to-groin cast suggested by Ponseti [1].

The second pillar of rotational control, which is friction to the shaft, is realized by a firm intake of the lower leg by using a closing cap at the level of the tibial tuberosity. Thereby and by using a three-point support the LLO is applying sufficient pressure on the bone and soft tissue to create enough friction to prevent slipping and rotation. In the shell-like slick plastic shaft of the classic AFO there is only few friction created by the punctual pressure of one or two Velcro straps bandings.

The orthotic design used by George et al. [22] consists of a sandal which is fixed to a longitudinal bar in external rotation (Fig. 7). The bar in turn is fixed to the shank by straps, holding the knee in constant flexion. Though being an interesting approach, the rotational control and in consequence stretching of the medial tissues of the foot was difficult to achieve and the deformity reoccurred in 31% of feet [22]. The authors supposed that the failing of the orthosis was due to full time knee flexion at 90° preventing active contractions of gastrocnemius and assumed that this in turn might result in tightness and a higher relapse rate.

Another feature of our articulated lower leg orthosis is the possible dorsiflexion of up to 20°. The dorsiflexion can be attained by active movement, by either walking in the orthosis or adding gas pressure springs. Thereby, the possibility to stretch and exercise the gastrosoleus complex as suggested by Desai et al. [27] is another advantage of the LLO design.

The efficacy of the Pohlig LLO in preventing clubfoot recurrence is difficult to assess in our retrospective study. Almost all the patients in the LLO group were secondarily provided with LLOs after failed FAO treatment; therefore, the whole group represents negatively selected cases.

Four children (one with bilateral and three with unilateral involvement) were treated with a lower leg orthosis from the beginning. All of them were pretreated. One of these children had received 9 months of casting elsewhere before being referred to us (unilateral clubfoot, Pirani score 3 at presentation). The parents were strongly inclined towards LLO. This approach failed, and the child had to be re-operated at the age of 7 years. The other three children (Pirani score, 4.5–6) were deliberately provided with LLO by the treating doctor who was at that time convinced that the LLO treatment was equally effective as the FAO. These three children showed no recurrence over a follow-up period of 8.1–9.5 years.

The children who were converted during treatment were treated with the LLO for a mean time of 31 months (subsequently to a mean treatment time of 8 months with a foot abduction brace), leaving a considerable amount of time for the LLO to ‘fail’. We did not detect an elevated percentage of recurrence in this group after a mean follow-up of 8.4 years.

Although we are satisfied with the compliance and efficacy of the Pohlig LLO, this system does have certain disadvantages. The orthoses can in fact be adjusted one or two times in size (by grinding down the Inliner and displacing the hinges that fix the foot-unit to the lower leg-unit), but it is still rather time-consuming to adjust the orthosis to the child’s growth. Another difficulty is that babies sometimes have a lot of soft tissue. This can limit the firm fitting and therefore reduces rotational stability and control of the foot. The orthosis cannot properly support itself to bony structures in these cases and instead ‘swims’ on soft tissue. Especially when a child has only a unilateral clubfoot, parents are often quite demanding to switch to a unilateral orthosis. If possible, we try to convince the parents to stick to the FAO until the age of at least one year before switching to an LLO. We feel that one year of age is likely the appropriate cut-off age for relevant technical problems. Nevertheless, if necessary, children below one year of age can be provided with an LLO, but it can be technically demanding and time consuming for the reasons noted above. In this age group, we now like to replace the lower leg unit by a thigh long L-shaped dorsal channelling with ventral cover in fixed 90° knee flexion. This unit is attached to the foot unit by screws in the same manner as in the LLO. The resulting construction strongly resembles the thigh long casting as performed in the Ponseti casting and provides – in our opinion - equivalent stability.

### Limitations of the study

There are several limitations to our study. First, the study is retrospective. Second, since most patients included in the study have been treated with a standard foot abduction brace before converting to the LLO, a comparison of the two methods is not possible and the statistical analysis is limited. Furthermore, the diagnosis of recurrence was established on a clinical basis. Because there were no objective measures in this decision (e.g. degrees of hindfoot-varus), there might be a selection bias. Another limitation is that the original work of Baise and Pohlig is only available in German language, which limits visibility and dissemination of this treatment option. Finally, the costs and efforts to construct a LLO are higher than purchasing a standard FAO from the shelf.

## Conclusion

Changing from FAO to LLO at any point during treatment did not result in an increased rate of surgery. The Pohlig LLO was associated with good compliance and efficacy in terms of recurrence of congenital talipes-equinovarus being treated with the Ponseti method. If treatment with a standard foot abduction brace cannot be continued, our results show that the LLO is an efficient alternative. This study is serving as a pilot for further investigations performed in a prospective and randomized manner.

## Additional files

Additional file 1: A video showing in full length how to put on the lower leg orthosis. (MP4 47817 kb)

Additional file 2: Raw data of our patients. (XLSX 22 kb)

## Abbreviations

FAO: Foot abduction orthosis
LLO: Lower leg orthosis
